# A novel multi-measure approach to study medical students’ communication performance and predictors of their communication quality - a cross-sectional study

**DOI:** 10.1186/s12909-025-07018-9

**Published:** 2025-05-09

**Authors:** Giulia Zerbini, Pia Schneider, Miriam Reicherts, Nina Roob, Kathrin Jung-Can, Miriam Kunz, Philipp Reicherts

**Affiliations:** 1 Department of Medical Psychology and Sociology, Institute of Theoretical Medicine, Faculty of Medicine, University of Augsburg, Stenglinstrasse 2, 86156 Augsburg, Germany; 2 Department of Medical Education Sciences (DEMEDA), Faculty of Medicine, University of Augsburg, Stenglinstrasse 2, 86156 Augsburg, Germany

**Keywords:** Doctor-patient communication, Non-verbal behavior, Standardized patient

## Abstract

**Background:**

Successful doctor-patient communication is a critical component of effective medical treatment and therefore plays a crucial role already in medical training. The aim of this cross-sectional study was to employ a multi-measure approach assessing different perspectives and variables to investigate how medical students’ communication performance differs across medical training (1st vs. 5th or 7th semester).

**Methods:**

43 medical students (1st semester: *N* = 23; 5th or 7th semester: *N* = 20) engaged in a simulated doctor-patient consultation with a standardized patient (SP) while being videorecorded. Medical students’ communication quality was assessed by a communication expert and by the SP (both being blinded regarding the semester of the medical student) using standardized questionnaires (Berliner Global Rating Scale, Medical Interview Satisfaction Scale). Following our multi-measure approach, we also assessed several non-verbal parameters and variables (back-channeling, turn-taking, verbal dominance, automatic analysis of emotional facial expressions, skin conductance level).

**Results:**

Analysis of non-verbal measures demonstrates that advanced students used more back-channeling, displayed more facial expressions of happiness and showed elevated skin conductance levels compared to 1st semester students. These non-verbal parameters could significantly predict the expert’s communication quality assessment, explaining 31% of the variance in communication quality. As expected, the expert and SP rated the communication quality of 5th /7th semester students as significantly better compared to 1st semester students.

**Conclusions:**

We found that non-verbal parameters significantly differed between students of early vs. later stages of their medical training and were predictive of communication quality. Especially, sympathetic responsiveness - likely indicating the level of involvement - correlated with expert evaluations. These findings suggest that effective communication becomes evident across different channels and that sincere engagement into a conversation might be a requisite for successful doctor-patient communication.

## Background

Good communication skills are essential to build a trustful and empathic doctor-patient relationship, which has long been recognized as an important determinant of patients’ health outcomes as well as patients’ satisfaction and adherence to therapies [1–3]. Teaching communication skills has thus become a central feature of medical degrees. At the Medical Faculty of the University of Augsburg, we have recently evaluated a newly developed communication curriculum (KomCuA) and could show that self-reported communication skills and attitudes towards empathy of higher-semester medical students are better compared to 1st semester students [4]. Our results are in line with several other studies showing that communication skills and empathetic behaviors of medical students usually improve when communication trainings are implemented in medical curricula, see for example the following reviews [5–8]. Previous studies have mainly used global measures of performance based on either self-reports (questionnaires) or observer reports (e.g. experts grading during objective structural clinical examinations – OSCEs) to evaluate students’ communication and empathetic skills [9–21]. Although such instruments represent valid and economic means to assess communication skills of medical students, they might overlook critical details, and are prone to certain biases such as social desirability (self-report) or halo effects (observer reports) [22]. Moreover, communication is a complex phenomenon comprising not only the spoken word, but also a variety of non-verbal channels and responses. Therefore, a multi-measure approach that covers multiple modalities and response systems in addition to holistic expert evaluations, might be ideally suited to study which communication features are crucial for effective communication and thus, might be addressed in medical training. Previous studies have already shown that non-verbal (re-)actions critically impact the quality of doctor-patient communication [23, 24]. Nevertheless, most studies so far incorporated only a limited number of variables, mainly focused on physicians rather than medical students and assessed communication performance in - low standardized - real-life settings.

To fill this gap, we here investigated medical students’ communication performance during a simulated consultation in a highly standardized setting, using a series of objective and reliable measures, which cover a variety of non-verbal aspects of doctor-patientcommunication, in addition to experts’ reports evaluating the communication quality more holistically. In our methodological approach, we incorporated guidelines by Asan and Montague regarding the use and analysis of videos when investigating complex interactions in health care contexts [25]. We decided on the assessment of physiological arousal referring to Hulsman et al. who recommend the assessment of physiological measures when investigating doctor-patient communication [26]. When selecting the non-verbal variables for the present study, we considered the Nonverbal Accommodation Analysis System by D´Agostino and Bylund [27], which covers behavior categories such as “talk time” and “smiling”; and further the article by Mast et al. [28], who investigated how patient satisfaction correlates with the physicians’ non-verbal facial responses, their non-verbal bodily and speech behaviors (for a complete list please see the respective articles). Accordingly, the following variables were selected: back-channeling, verbal dominance, turn-taking, emotional facial expressions and skin conductance. Back-channeling describes signs of reception and belongs to a set of essential communications techniques referred to as active listening, which were found to positively affect doctor-patient communication [6, 23, 29]. A balanced participation of doctors and patients during consultations is also a critical aspect impacting patients’ satisfaction [30], which can be quantified by assessing verbal dominance that is the relative time of speech during a consultation. Relatedly, switching between talking and listening in a conversation (turn-taking) is an important feature of effective doctor-patient communication, increasing comfort and securing information transfer [31]. Furthermore, we analyzed students’ facial expressions, given that facial expressions represent probably the most crucial non-verbal communicative channel in (teaching) doctor-patient communication [32] and especially the role of smiling in healthcare context has just recently been highlighted [33]. Our multi-measure approach was complemented by assessing an index of physiological arousal, namely skin conductance level (SCL). Changes in SCL reflect sympathetic activation indicating the preparation of the organism for action [34]. The assessment of SCL expands previous research on doctor-patient communication, which mainly focused on correlates of intense stress responses like endocrine activation [35].

In summary, the aim of the present study was (i) to employ a multi-measure approach to study communication performance of 1st vs. higher semester medical students during a highly controlled and standardized simulated consultation and (ii) to investigate which of the non-verbal variables might best predict communication quality (SP and expert ratings).

## Methods

The present cross-sectional study took place during the winter semester 2022/2023 (cross-sectional design). Data were collected in the laboratory of the Department of Medical Psychology and Sociology, Faculty of Medicine, University of Augsburg.

### Participants

Medical students at the University of Augsburg enrolled in the 1st semester and 5th or 7th semester were recruited via internal emails and postings. A total of 45 students (23 enrolled in the 1st semester and 22 enrolled in 5th /7th semester) agreed to participate in the study. Students at the Medical Faculty in Augsburg follow a structured curriculum teaching communication (see [4]). The essentials of doctor patient communication (theory, communication tools and practice with simulated patients) are extensively taught during the first 4 semesters (5 lectures, 7 seminars, 4 small group tutorials with SPs, including being examined during an OSCE). Thus, after completion of the 4th semester, medical students have acquired a solid level of communication competency. For this reason and given the smaller sample size of 5th and 7th semester students, we decided to merge those two semesters for further analyses. All participants gave their informed consent and could choose whether they preferred to receive course credit or a monetary compensation for their participation. To be eligible for data analysis, advanced students of 5th /7th semester must have attended at least 25% of the structured communication curriculum thus far, to provide sufficient specificity regarding group allocation and between factor analyses. This criterion affected two participants. Skin conductance data of one participant (5th /7th semester) could not be analyzed due to technical failure during recording and was thus excluded from further statistical analyses. Descriptive data of the final sample can be found in Table 1.

### Simulation of a doctor-patient consultation

**Fig. 1:**
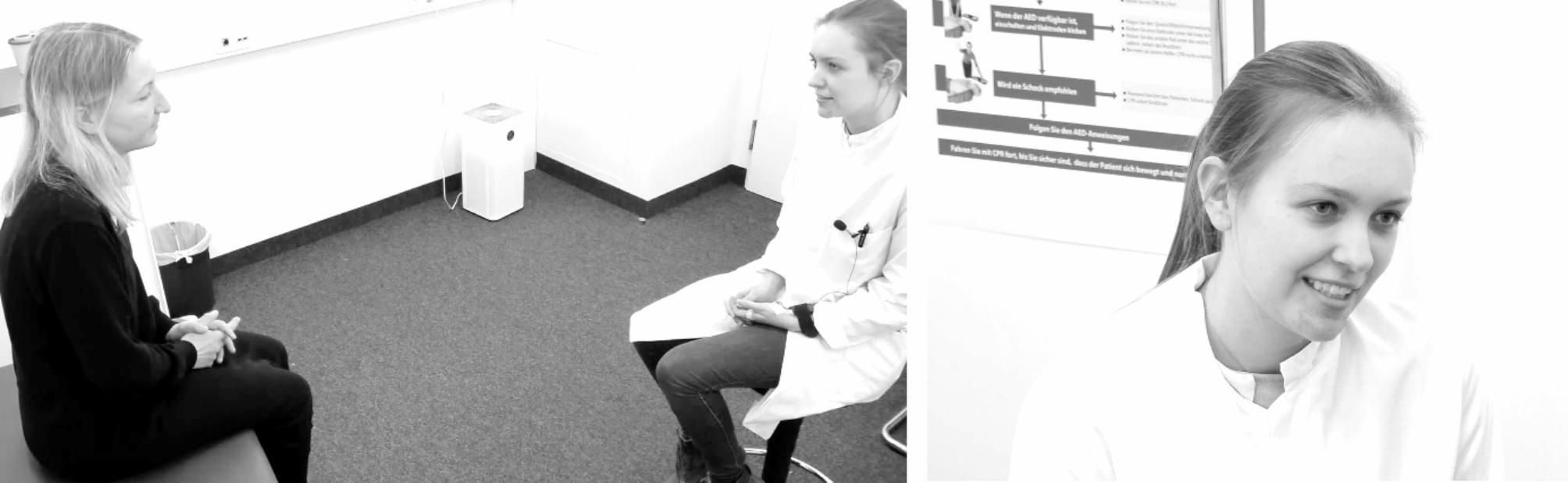
Consultation room with the SP and a medical student. The left video perspective was used by the expert to rate the performance of the medical student. The right video perspective was used for automatic analysis of the emotional facial expression of the medical student

The simulated consultation took place in a room (the same for all students) of our laboratory that is equipped with high-resolution video-cameras and had been furnished to resemble a doctors’ consultation room (including a patient examination table, medical devices). Students had 15 min time to read the case narrative: A female patient asks for advice after a tick bite. The tick has been removed, but the patient worries about transmission of diseases and thus, asks for medical advice. All the necessary medical information was summarized in a concise, slightly simplified way in order to enable the 1st semester students to successfully complete the consultation without prior knowledge. Moreover, all students were instructed to talk with the patient about their worries in an empathetic way. Scenario, setting, and the simulated patient (SP) were highly standardized and the same for all students (one SP played the role for all medical students). The students had about 7 min to talk to the SP and were informed that at the end of the 7 min the experimenter (sitting in an adjoining room and controlling the video recordings) would inform them to conclude the consultation. The consultation was video recorded for later analyses (see Fig. 1).

### Multi-measure assessment of communication performance

#### Observer reports (assessment of communication quality)

##### Expert ratings

One independent expert, who was blinded to the semester, in which students were enrolled, rated the videos regarding the performance of the students using the Berliner Global Rating Scale (BGR; [36]. The BGR is a German translation of the Analytic Global Scale [37] and is frequently used to evaluate the quality of doctor-patient communication, especially in training contexts. The scale comprises 4 items: empathy, coherence, verbal, and non-verbal communication. Each item is assessed on a 5-point Likert scale, which aligns with the German Grading System, with the number 1 being the best grade (equaling an “A” or a grade point average of GPA = 4) and the number 5 being the lowest grade (equaling an “F” or a GPA = 0). At both ends of the scale, descriptions of positive and negative examples are provided. An average BGR score was computed across the 4 items. The expert is a certified psychotherapist with longtime experience in training the instructors and lecturers who teach the communication classes at our Medical Faculty. Another observer (a psychologist and communication teacher) independently rated 15% of the videos (three from 1st semester students and three from 5th /7th semester students) to calculate the interrater reliability. Intraclass Correlation Coefficient (ICC, Two-Way Mixed Model, evaluation of consistency for single measures) was calculated between the two experts and a good agreement was found (ICC_BGR_=0.80).

##### SP ratings

The Medical Interview Satisfaction Scale (MISS-21), which has been shown to have good psychometric properties [38], was used to assess SP´s satisfaction with the medical consultation. We used a shortened version with 7 items of the “rapport” and “communication comfort” subscales (items Nr. 6,8,9,10,11,12,14) which were more suitable for our simulated medical consultation (e. g. “The doctor gave me a chance to say what was really on my mind.”). Each item was rated on a 7-point Likert scale and a mean score was calculated, with higher values indicating more satisfaction.

#### Non-verbal parameters

The simulated consultations were all video-recorded using a high-quality video recording system (2 cameras Panasonic AW-HE40SWEJ; 1 video recorder PEARL-2 Epiphan System Inc.; 2 microphones Audio Technica U 853 AW; 1 audio processor dbx ZonePro 1260 M, see Fig. 1). The following parameters were extracted from the videos: back-channeling, turn-taking, verbal dominance and emotional facial expressions. Using the Observer software (Noldus, Wageningen, NL) coders annotated on- and off-set of speaking segments and coded back-channeling.

*Back-channeling* refers to verbal markers uttered by the listener to indicate sustained attention and attentive listening (e.g. “hmm”, “OK” “uh huh”) [39]. They serve the purpose to keeping up the communication flow by encouraging patients to continue talking by minimal verbal prompts. The number of times that medical students used back-channel responses was annotated, later extracted and counted (summed) for each medical student. To ensure reliability, 10% of the video recordings were annotated by a second coder (in line with coding of non-verbal behavior [40]) and intraclass correlation of ICC = 0.94 indicated excellent reliability.

*Turn-taking* refers to the number of times that the roles of speaker and listener changed during the 7 min conversation. We annotated on- and off-set of speaking segments for the medical students and the SPs (this was performed by one coder) and computed the number of turn-takings that occurred during the whole consultation based on these annotations.

*Verbal dominance* refers to the ratio between the speaking time of medical students compared to SPs. This was computed by dividing the total length (sum) of the medical students’ speaking segments by the SPs total length (sum) speaking segments, with values above 1 indicating higher verbal dominance of the students.

##### Emotional facial expressions

The videos were automatically analyzed using the software FaceReader from Noldus (Wageningen, NL) to assess the students’ emotional facial expressions. The FaceReader has been shown to validly detect various emotional facial expressions, especially with regard to the detection of happiness expressions [41]. With the FaceReader software each frame was categorized into one of 6 different basic emotions, namely happy, sad, angry, surprised, scared, disgusted and a neutral state. If the software was unable to detect any of the emotional or neutral states, the respective frames were labeled “Unknown”. In a second step the sum of frames per emotional category was calculated and the ratio of each emotion by the sum of all frames, minus the sum of “Unknown” frames, was calculated, representing the relative length of each identifiable response category, ranging from 0 to 1. Subsequently, facial response categories whose relative length exceeded a criterion of 0.05, i.e. 5% of identifiable frames, were selected for further statistical analysis, in accordance with previous studies and considerations regarding the analysis of facial responses, see for example [42, 43]. For further details regarding the software and algorithms, please see [44].

##### Physiological arousal

Students’ electrodermal activity (EDA) was continuously recorded via a wireless amplifier and data storage (BN-PPGED; Biopac Systems, Goleta, CA) using a pair of Ag-AgCl electrodes (0.8-cm diameter) that were attached to the inner palm of the left hand and the application of 0.5 V. The EDA signal was sampled at 125 Hz and the raw signal was further processed using Ledalab, version 3.4.9 [45]. After downsampling the signal to 20 Hz, the mean skin conductance level (SCL) across the whole period of the conversation (7 min) was calculated and analyzed statistically.

### Questionnaires

Given that previous studies have shown that personality traits, such as openness, agreeableness and conscientiousness are related to more positive attitudes towards the importance of communication and empathy as well as to better self-reported communication skills [46], we asked the students to fill out the BFI-K (Big Five Inventory short form; BFI-K; [47]) before they came to the laboratory for the simulated consultation. The BFI-K has 5 subscales (extraversion, agreeableness, conscientiousness, openness to experience, neuroticism). We additionally assessed social anxiety using the SPAI (Social Phobia and Anxiety Inventory; [48]).

### Statistical analyses

Communication performance (observer reports and non-verbal parameters, except for facial - emotional - responses) was compared between 1st semester and 5th /7th semester students using two sample t-tests. The significance level was set at α < 0.05. In case homogeneity of variance was violated (Levene Test), corrected *p*-values (Welch Test) are reported. In addition to *p*-values, Cohen’s *d* effect sizes [49] are reported. Emotional facial expressions were compared between 5th /7th semester using a Multivariate Analysis of Variance including happy and neutral expressions, which were followed up by univariate ANOVAs for happy and neutral expressions, separately. Partial eta squared is reported as indicator of effect size. Moreover, linear regression analyses were performed to investigate which of the non-verbal parameters can predict communication quality (observer reports) best. Statistical analyses were performed using SPSS 28 (IBM statistics).

## Results

### Demographics

1st semester and 5th /7th semester students did not statistically differ from each other in terms of sex ratio or personality traits that have been shown to impact communication performance (see Table 1). Although 5th /7th semester students were significantly older than 1st semester students, the mean difference was only two years.

**Table 1:** Sample characteristics of 1st and 5th /7th semester students

	1st Semester *(N = 23)*		5th /7th Semester *(N = 20)*		Group-differences (*p*-values)
Sex (female/males)	(10/13)		(14/6)		.125^a^
	*M*	*SD*	*M*	*SD*	
Age in years	21.17	2.8	23.15	2.8	0.025*^b^
BFI-K Extraversion	3.51	0.9	3.66	1.1	.625^b^
BFI-K Agreeableness	3.33	0.7	3.60	0.8	.251^b^
BFI-K Conscientiousness	3.82	0.6	3.79	0.6	0.877 ^b^
BFI-K Neuroticism	2.97	0.8	2.96	0.9	.985^b^
BFI-K Openness to Experience	3.77	0.8	3.67	0.9	.697^b^
SPAI Sum Score	42.93	19.3	40.24	19.4	.137^b^

Note: ^a^chi-square test was used for group comparison; ^b^t-tests were used for group comparisons, *p <.05

BFI-K: Big Five Inventory short form (German version); SPAI: Social Phobia and Anxiety Inventory

BFI-K scores range between 1–5 with higher values indicating higher degrees of extraversion, agreeableness, conscientiousness, neuroticism and openness. SPAI score ranges between 0-132, with higher values indicating more social phobia and anxiety symptoms

### Multi-measure assessment of communication performance

#### Observer reports (assessment of communication quality)

##### Expert report

**Fig. 2:**
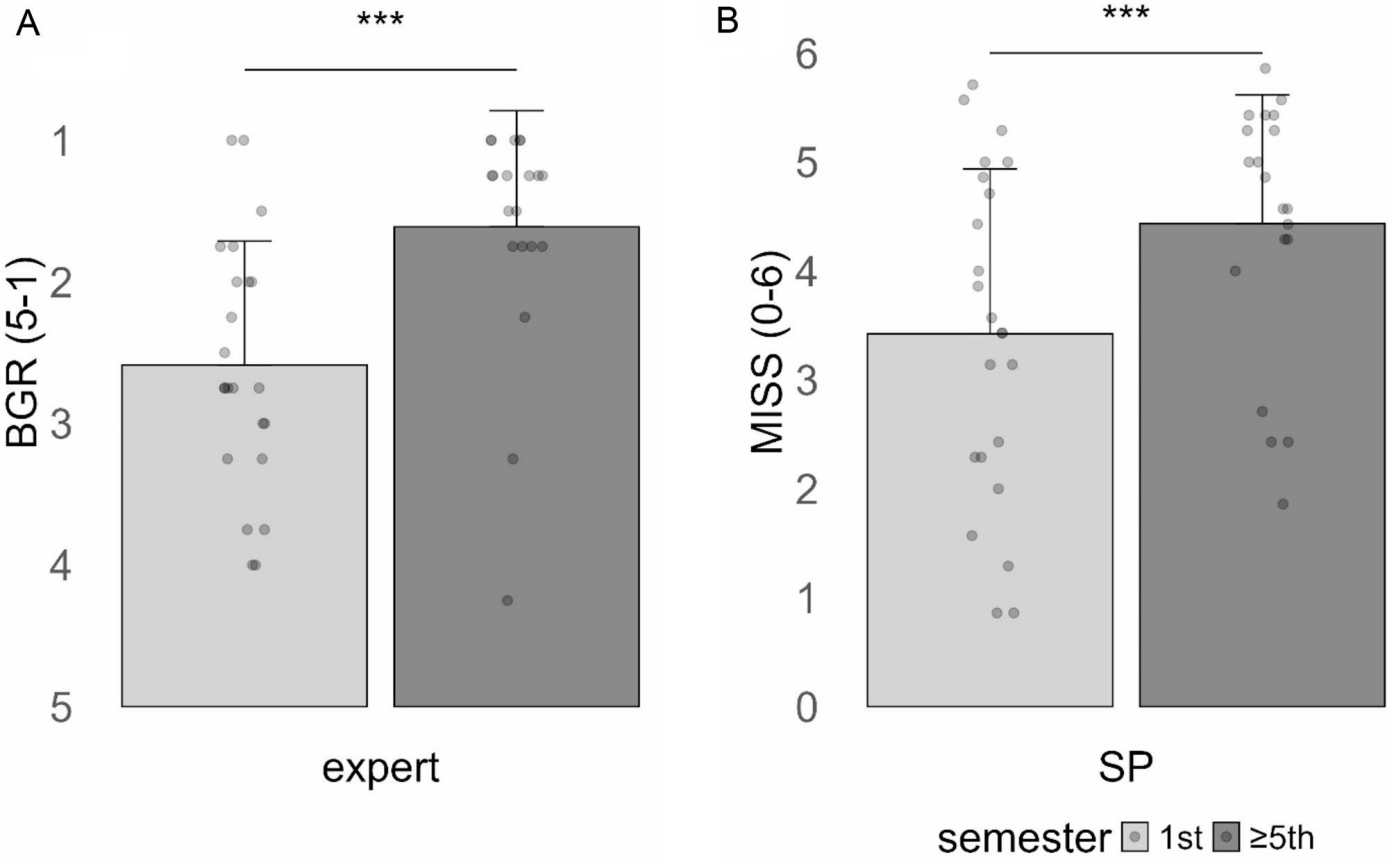
Observer reports (mean and SD) of the communication quality. Values are given separately for the Expert and the Simulated Patient (SP) ratings as well as separately for 1st and 5th /7th semester students. *Note: BGR: Berlin Global rating scale*, MISS: Medical Interview Satisfaction Scale; SD: standard deviation. ***p <.001

As can be seen in Fig. 2_A_, the communication expert (who was blinded with regard to the semester of the medical students) rated the communication quality of 5th /7th semester students as significantly better compared to 1st semester students (*t* [41] = 3.75, *p* <.001, Cohen’s *d* = 1.15). When comparing each of the 4 BGR items between 1st and 5th /7th semester students, it became apparent that advanced students showed better performances (large effect sizes) across all subscales (empathy *d* = 0.93, coherence *d* = 1.13, verbal communication *d* = 0.99, and non-verbal communication *d* = 1.04).

##### SP report

As can be seen in Fig. 2_B_, the SP (who was also blinded with regard to the semester of the medical students) gave significantly higher satisfaction / comfort ratings for consultations with 5th /7th semester students compared to 1st semester students (*t* [41] = 2.42, *p* =.020, Cohen’s *d* = 0.74).

#### Non-verbal parameters

##### Back-channeling

**Fig. 3:**
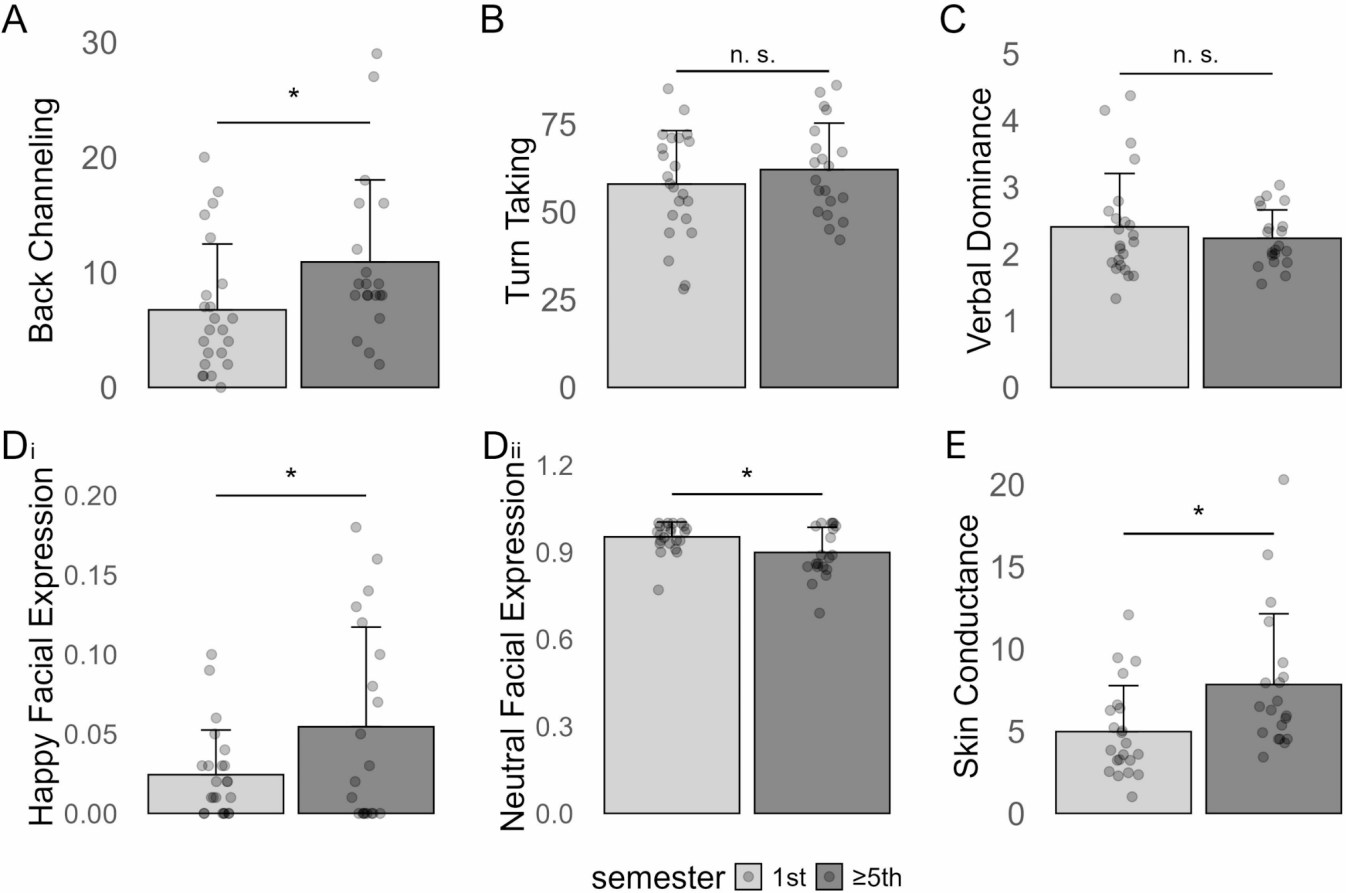
Non-verbal parameters (mean and SD) assessed during the medical consultation. Values are given separately for 1st and 5th /7th semester students. **A**) Number of back-channeling events; **B**) Number of turn-taking events; **C**) Ratio of speech duration of students > SP; **D**_**i**_) Ratio of happy expressions relative to the total of identified expressions; **D**_**ii**_) Ratio of neutral expressions relative to the total of identified expressions; **E**) Skin conductance in µS. Note: SD: standard deviation; *p <.05

Across the approximately 7 min of consultation an average of 8.7 back-channel responses were shown by the medical students (across all semesters). As can be seen in Fig. 3_A_, more back-channeling was found in 5th /7th semester students compared to 1st semester students (*t* [41] = 2.12, *p* =.040, *d* = 0.65).

##### Turn-taking

Across the approximately 7 min of consultation an average of 59.8 communication turns (speaker and listener role changing between medical students and SP) occurred. When comparing turn-taking between semesters, no significant difference was found (*t* [41] = 0.94, *p* =.351, *d* = 0.29) between 1st and 5th /7th semester (see Fig. 3_B_).

##### Verbal dominance

Medical students spoke more (on average for 5:05 min, SD = 38 s.) compared to the SP (on average for 2:03 min, SD = 33 s.) during the consultation, thus resulting in a mean verbal dominance score of 2.3 for the medical students. As can be seen in Fig. 3_C_, verbal dominance was not significantly different between 1st and 5th /7th semester (*t* [41] = 0.86, *p* =.397, *d* = 0.26).

##### Emotional facial expressions

Automated decoding of facial expressions performed with the Noldus face reader software revealed that besides a neutral expression, only the expression of happiness was shown more than 5% of the time during the conversation (corrected by the length of not identifiable expressions). Multivariate analysis, including happy and neutral facial expressions, demonstrated a significant group factor, *F* [2, 40] = 3.39, *η*_*p*_ = 0.15. Univariate ANOVAs revealed a significantly higher proportion of neutral expressions in 1st semester students compared to 5th /7th semester students, *F* [1, 41] = 6.25, *p* = 0.02, *η*_*p*_ = 0.13. For happy expressions the opposite was true, that is a higher proportion of happy expressions for 5th /7th semester students compared to 1st semester students, *F* [1, 41] = 4.38, *p* = 0.04, *η*_*p*_ = 0.10, see Fig. 3_Di_ and 3_Dii_.

##### Physiological arousal (skin conductance)

As can be seen in Fig. 3_E_, significantly elevated skin conductance levels were found in 5th /7th semester students across the whole consultation compared to 1st semester students (*t* [40] = 2.58, *p* =.014, *d* = 0.80). Thus, advanced medical students showed higher physiological arousal during the consultation.

### Prediction of communication quality

In a last step, we analyzed whether the non-verbal parameters could predict communication quality (observer report) and which parameters might be the best predictors. To this aim, regression analyses were performed separately for the expert ratings (BGR) and the SP ratings (MISS-21). The non-verbal parameters could significantly predict BGR ratings of the expert (*F* [6] = 2.56, *p* =.037; *r*^*2*^ = 0.31), explaining 31% of the variance. Standardized beta values showed that especially the physiological arousal (skin conductance level) significantly predicted experts BGR ratings (beta = − 0.345; *p* =.037), with higher arousal predicting better communication quality. None of the other beta values (back-channeling (beta = − 0.044) turn-taking (beta = 0.04) verbal dominance (beta = 0.317), neutral facial expressions (beta = 0.169), happy facial expressions (beta = − 0.121)) reached significance (all p-values ≥ 0.05).

In contrast, SP ratings (MISS-21) could not be significantly predicted by the non-verbal parameters (*F* [5] = 1.64, *p* =.174; *r*^*2*^ = 0.186).

## Discussion

In the present study we used a multi-measure approach to investigate how communication quality differs between early and advanced medical students. In addition to standardized scales, we assessed back-channeling, turn-taking, verbal dominance, the students’ (emotional) facial responses and their skin conductance level.

### Standardized assessment of communication quality in medical students

Students from higher semesters showed better communication skills during the consultation as indicated by the ratings provided by the SP and by the expert. This finding might not be surprising given that at the time of the study, 1st semester students had not yet attended any classes of our longitudinal communication curriculum (KomCuA; [4], while 5th /7th semester students had already successfully completed all basic communication trainings. Our findings are also in line with several other studies that have shown how communication skills of medical students improve across the medical degree, when a communication curriculum is implemented [4–8, 10–13, 15, 16].

As already outlined, the majority of previous research on communication progress of medical students used self-report ratings or grades obtained during OSCEs. While the last should provide a more objective way of assessing communication performance, the content of the OSCEs might vary regarding difficulty and complexity, when comparing students enrolled in different semesters. Thus, students’ grades might not be directly comparable. Moreover, different examiners usually grade the students during OSCEs, which could additionally influence the results, given that a certain degree of inter-rater variability has been already reported [50, 51]. Finally, assessing communication skills during OSCEs can be confounded by the exam situation, which might be experienced as more or less stressful by the students and in turn could differently impact their performance [52]. The motivation of the present study was to address these limitations by (a) assessing the students’ communication skills outside of an examination context, (b) using the same standardized simulated consultation, (c) providing the students with the necessary medical information in an easy and concise way to ensure comparable conditions for all students, and (d) by having only one SP and one expert - both being blinded with regard to the semester of the students - evaluating all consultations. Altogether, our findings confirm the results from previous studies [4–8, 10–13, 15, 16] and demonstrate improved communication performance in medical students after attending carefully designed communication courses.

### Non-verbal measures of doctor-patient communication

We found that certain non-verbal aspects of communication varied significantly between students from different semesters. The importance of non-verbal communication is typically emphasized in medical communication curricula. For instance, when communication techniques related to active listening and empathy are taught and trained, non-verbal (e.g., body lean and eye contact, back-channeling) aspects of communication are emphasized [53]. The simulated doctor-patient consultation in the present study was kept quite simple in order to allow also the 1st semester students to successfully complete the assignment. Given the standardized laboratory setting and the lack of (medical) complexity of the consultation, we focused on non-verbal responses that could actually vary in the simulated communication context of the present study, ignoring otherwise critical behaviors such as “not writing during consultation” or the “seating position relative to the patient”. We found that higher semester students showed increased sympathetic activation, increased facial expressions of happiness and a higher rate of back-channeling compared to 1st semester students. The finding that advanced students used more back-channel prompts and displayed more friendly (happy) facial expressions was expected, since the importance of active listening is stressed out in our communication curriculum, according to the notion that active listening increases patients’ satisfaction [29]. In semester two and three of our communication curriculum [4] we focus on non-verbal communication and train the technique of active listening with the students. Moreover, these non-verbal aspects are also tested in an OSCE after semester 3. Given that it has been shown that active listening is a trainable skill [54], we also expected to find elevated rates of active listening in higher semester students. These results are in line with previous studies highlighting the importance of training non-verbal communication among medical students [55–58]. Students that use non-verbal communication to convey interest and empathy (e.g., by using facilitative nodding) usually receive higher ratings from SPs evaluating their satisfaction during the consultation, and also from experts evaluating the quality of the consultation [55]. Previous studies have however evaluated non-verbal communication by analyzing the video-taped consultations using standardized checklists describing different non-verbal behaviors [55–58], whereas studies using other methods to assess non-verbal communication, such as the ones employed in the current study, are still lacking.

Regarding the skin conductance level, we interpret increased sympathetic activity in advanced students as indexing elevated task engagement, which was correlated with better communication performance. This is in line with previous findings by Meunier et al. [59], who found a positive association between physiological arousal and communication performance in a breaking bad news scenario. The opposite results, namely lower arousal in higher semester students could have also been expected, given that higher semester students are more trained and experienced and thus, possibly less stressed during the consultation, in accordance with earlier research that found longer job experience being associated with lower arousal levels during stressful and difficult medical consultations [60]. However, in the present study we intentionally decided against a highly stressful and technically demanding consultation, choosing a straightforward task i.e. informing the patient about ticks, so that 1st semester students might not feel overwhelmed and to prevent negative affect due to failure. Moreover, the task was designed to provide many possibilities for the students to guide the conversation. Therefore, we are more inclined to interpret higher SCL as indicative for advanced students being more engaged in the consultation and thus, more successful than 1st semester students, in line with previous studies, demonstrating a positive association of sympathetic activation, mental effort and performance in diverse cognitive tasks [61–63].

Surprisingly, we did not find significant group differences regarding the number of times that the roles of speaker and listener changed between the SP and the students (turn-taking) and with regard to the students’ relative speaking time (verbal dominance). Previous studies have shown that turn-taking is positively associated with patient satisfaction and patient centeredness and more collaborative decision-making [31, 64]. We thus assumed that more turn-taking would indicate a more balanced and effective consultation and would increase across semesters. However, the frequency of turn-taking did not vary between semesters, which could be explained by the relatively short duration of the consultation (7 min) not allowing for enough variation in this variable. Turn-taking so far has not been investigated in the context of medical education, except for one study investigating the role of feedback in teaching students about their individual pattern of non-verbal communication [65], accordingly more research is clearly warranted. Students’ verbal dominance also did not vary between semesters. Overall, we found that the medical students (regardless of the semester) verbally dominated the consultation. This is in line with a previous study that also found that medical students verbally dominated the consultation in a standardized doctor-patient interaction and more importantly, also found that verbal dominance did not differ between medical students with good and poor overall communication skills [66]. Thus, verbal dominance might show less variance in simulated consultations, although this variable seems to be an important factor for patient satisfaction and enablement in everyday doctor-patient consultations [24], given that verbal dominance may determine the character of the doctor patient relationship (e.g. paternalistic vs. participative) [67].

When investigating our non-verbal parameters as predictors of communication quality (as rated by the expert across all medical students), 31% of the variance was explained by the regression model. Amongst these predictors, it was especially the physiological arousal that significantly predicted the communication quality. In line with our interpretation of the increased SCL across semesters, this finding indicates that greater engagement in the consultation leads to better communication performance. The other predictors did not reach significance, which might be due to the sample size and interrelatedness of the variables. Clearly, future replication of the identified predictors is necessary, employing large and - with regard to communication competencies - heterogeneous samples.

### Limitations

In the present study we infer that communication performance improved due to participation in the medical communication curriculum by comparing different semesters cross-sectionally, however longitudinal designs are better suited to provide conclusions based on causality. Future research including repeated measures, closely following the students’ progress, is warranted. Here, we decided on a set of crucial non-verbal variables but of course the selection is not conclusive. The incorporation of more diverse measures and variables, especially regarding physiological responsiveness (see also: [26]), is a challenge for future research. Furthermore, replication of the present findings especially regarding the predication of communication performance in larger samples is needed. Nonetheless, post hoc power analysis performed with g*power [68] for the group comparison regarding BGR expert ratings revealed a satisfying test power of 1-beta = 0.96.

## Conclusions

Using a multi-measure approach, we could demonstrate differences in non-verbal communication parameters comparing early and advanced medical students. Especially, emotional responses, back-channeling and sympathetic responsiveness - likely indicating the level of engagement - differed across semesters and were predictive of better communication quality. Verbal dominance and turn-taking did not significantly discriminate between semesters, likely due to the characteristics of the highly standardized consultation and the nature of the task, leaving only little space for variation. These findings suggest that effective communication becomes evident across different channels and that sincere engagement into a conversation might be a requisite for successful doctor-patient communication.

## Data Availability

Data is available upon request, except for the video recordings.
